# Anesthetic and Transfusion Management in Placenta Accreta Spectrum: Lessons From a Resource-Limited Setting and Mini-Review

**DOI:** 10.14740/jmc5204

**Published:** 2025-12-24

**Authors:** Alma Soxhuku Isufi, Genci Hyska, Kastriot Dallaku, Vjollca Shpata, Xhensila Frasheri Prendushi, Albana Shahini, Asead Abdyli, Krenar Lilaj, Hektor Sula, Rudin Domi, Fatos Sada

**Affiliations:** a Service of Anesthesia and Intensive Care, University Hospital Center “Koco Gliozheni”, Tirana, Albania; b Department of Rehabilitation, Faculty of Rehabilitation Sciences, Sports University of Tirana, Tirana, Albania; c Department of Clinical Sciences, Faculty of Rehabilitation Sciences, University of Medicine, Tirana, Albania; d Department of Diagnostics and Rehabilitation, Faculty of Rehabilitation Sciences, University of Medicine, Tirana, Albania; e Department of Anesthesiology and Intensive Care, American Hospital 3, Tirana, Albania; f Department of Surgery, Service of Anesthesia and Intensive Care, University of Medicine, Tirana, Albania; g Department of Anesthesiology and Reanimation, Faculty of Medicine, University of Pristina, Pristina, Kosovo

**Keywords:** Placenta accreta spectrum, Anesthesia, Cesarean section, Massive bleeding, Massive transfusion protocol

## Abstract

Placenta accreta spectrum (PAS) is a severe obstetric condition characterized by abnormal placental invasion of the myometrium, often resulting in massive hemorrhage and high maternal morbidity and mortality. Optimal management requires early recognition, multidisciplinary coordination, and prompt activation of massive transfusion protocols (MTPs). We report the case of a 41-year-old gravida 3 woman at 36 - 37 weeks of gestation, with two prior cesarean deliveries and a transverse fetal lie, who developed life-threatening hemorrhage during cesarean section for PAS. Spinal anesthesia was promptly converted to general anesthesia to allow safe surgical intervention, which included hysterectomy, hemostatic and vaginal sutures, bladder repair, and massive transfusion. Postoperatively, the patient was stabilized in the intensive care unit and discharged in good condition after 10 days. This case demonstrates that early MTP activation, rapid anesthetic adaptation, and coordinated multidisciplinary care can result in favorable outcomes even in resource-limited settings. It underscores the importance of preparedness, flexible intraoperative decision-making, and collaboration across obstetric, anesthetic, surgical, and critical care teams in the management of high-risk PAS cases.

## Introduction

Placenta accreta spectrum (PAS) disorders are among the most serious complications in modern obstetrics, caused by abnormal trophoblastic invasion into the myometrium and failure of normal placental separation [1]. Their incidence has risen alongside increasing cesarean deliveries, now estimated at 1 in 333 - 533 births [2]. Major risk factors include placenta previa, prior cesarean section, advanced maternal age, multiparity, and assisted reproduction [3, 4]. PAS is classified as accreta, increta, or percreta, with reported distributions of 63%, 15%, and 22%, respectively [2].

PAS is often complicated by life-threatening hemorrhage and peripartum hysterectomy, with outcomes worsening when diagnosis is delayed or resources are limited [1, 5]. Although multidisciplinary management is recommended, practical implementation can be difficult in low-resource settings lacking blood products, interventional radiology, or surgical expertise [5].

This report highlights the challenges of managing massive hemorrhage due to PAS in a resource-limited setting, underscoring early massive transfusion protocol (MTP) activation, timely anesthetic conversion, and coordinated multidisciplinary care to achieve a favorable outcome.

For this mini-review, we performed a comprehensive search of PubMed and Scopus (January 2000 to January 2025) using MeSH terms and keywords: “placenta accreta spectrum,” “anesthesia,” “cesarean section,” “massive bleeding,” and “massive transfusion protocol.” Eligible studies included human research in English reviews, randomized trials, observational studies, and editorials. Non-English, animal, non-peer-reviewed, or inaccessible articles were excluded. Two authors (RD and ASI) screened titles and abstracts, with 39 full texts included for review. Ethical approval was waived according to institutional policy for single case reports.

## Case Report

### Investigations

**Figure 1:**
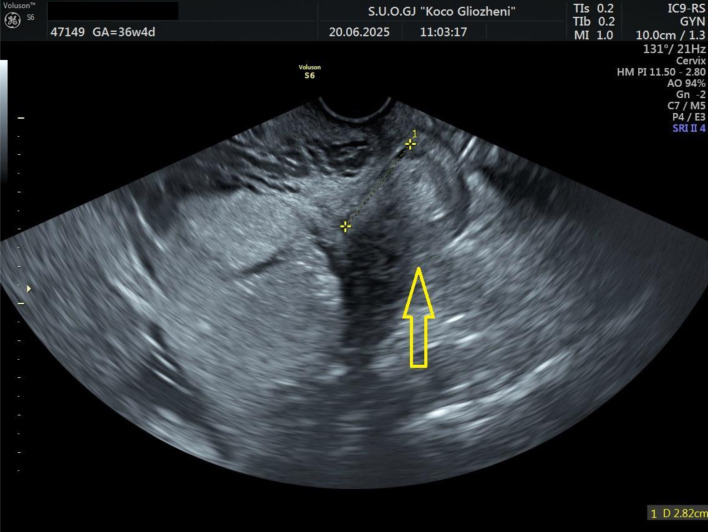
Ultrasound examination showing placental tissue invading the lower myometrium (arrow).

**Figure 2:**
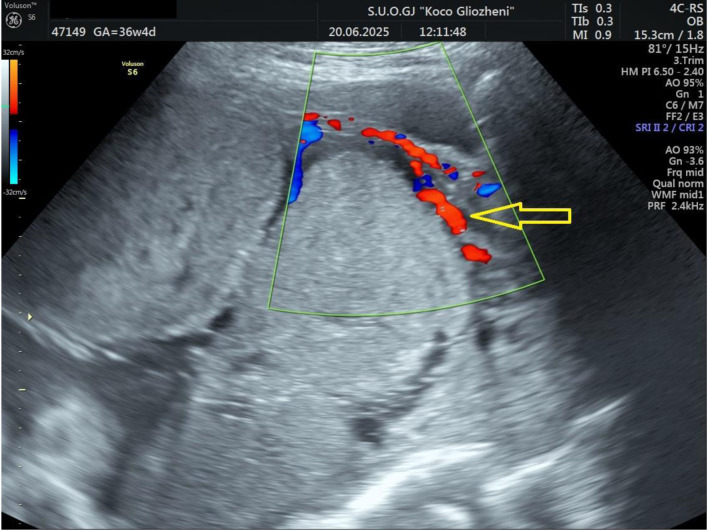
Doppler ultrasound examination showing placental tissue invading the myometrium and extending to the serosa. Increased vascular flow was demonstrated on color Doppler ultrasound (arrow).

A 41-year-old woman at 36 - 37 weeks’ gestation, body mass index (BMI) 26 kg/m^2^, with a history of two prior cesarean deliveries and a transverse fetal lie, was admitted for evaluation. Ultrasound showed an anterior placenta completely covering the internal os, invading the full myometrial thickness up to the serosa, consistent with PAS, with marked Doppler vascularization (Figs. 1 and 2). So, elective cesarean section was indicated, and the blood bank was notified ensuring blood reserves. The surgical team was composed by obstetrician, vascular surgeon, and urologist.

Cesarean section was initiated under spinal anesthesia. After neonatal delivery, manual placental separation caused massive hemorrhage, prompting endotracheal intubation and conversion to general anesthesia. Abdominal hysterectomy was performed, with additional hemostatic sutures, bladder repair by a urologist, and vaginal hemostasis by a vascular surgeon. Bleeding was controlled after about 3 h. The baby was delivered uneventfully with an Apgar score of 9 and a birth weight of 2,725 g.

### Treatment

Initial management included spinal anesthesia and crystalloids via two large-bore intravenous (IV) lines (blood pressure (BP) 110/55 mm Hg, heart rate (HR) 75 bpm). Following placental separation, hypotension (BP 50/35 mm Hg) and tachycardia (HR 78 - 135 bpm) required general anesthesia. Central venous and arterial lines were placed; norepinephrine infusion (0.05 - 0.2 µg/kg/min) maintained mean arterial pressure (MAP) ≈ 60 mm Hg. An MTP was activated, delivering 14 packed red blood cells (PRBCs), 10 fresh frozen plasma (FFP), 12 platelets, 3.5 L of crystalloids, and 2 g tranexamic acid (TXA), with active prevention of hypocalcemia and hypothermia.

### Follow-up and outcomes

**Figure 3:**
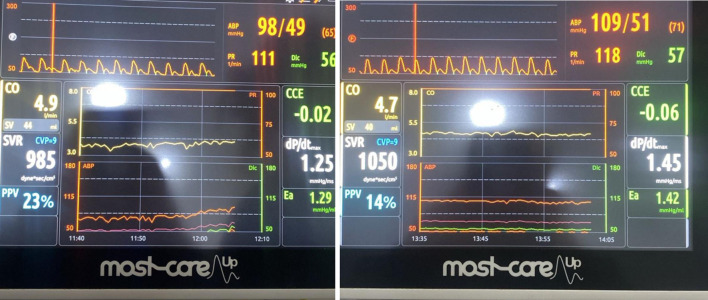
Most-Care monitor in intensive care unit.

Surgery lasted 4.2 h with an estimated 4 L blood loss. The patient remained hemodynamically stable and was transferred to the intensive care unit (ICU) for monitoring with the Most-Care system (Fig. 3).

The cardiac output (CO) was within normal limits; however, a pulse pressure variation of 23% suggested significant hypovolemia, indicating the need for additional fluid resuscitation and vasopressor support to maintain hemodynamic stability. Both CO and stroke volume (SV) were at the lower end of the normal range, consistent with reduced preload and ongoing volume deficit.

Postoperatively, she was alert and stable (BP 90/45 mm Hg, HR 110 bpm); labs showed Hb 6.5 g/dL, platelets 75 × 10^3^/µL, fibrinogen 223 mg/dL, pH 7.21, base excess (BE) -14.1, and lactate 5.6. Supportive therapy included two blood units, albumin, antibiotics, electrolytes, and anticoagulants. She was discharged in good condition after 10 days.

## Discussion

PAS is a major obstetric complication involving abnormal placental invasion, leading to retained placenta, massive hemorrhage, and high maternal morbidity. We report a 41-year-old gravida 3 at 36 - 37 weeks with two prior cesareans and a transverse lie who developed life-threatening bleeding during cesarean delivery for PAS. Management included conversion to general anesthesia, hysterectomy, bladder repair, hemostatic suturing, and massive transfusion, emphasizing the importance of timely decision-making and multidisciplinary coordination.

PAS poses a major clinical challenge due to abnormal placental invasion into the myometrium and, in severe cases, adjacent pelvic structures. It is commonly associated with massive hemorrhage, transfusion requirements, urologic or gastrointestinal injuries, and prolonged ICU admission, contributing to high maternal morbidity and mortality [6]. Table 1 summarizes the complications associated with PAS.

**Table 1:** Complications Associated With PAS

Category	Complications
Hemorrhagic	Massive obstetric hemorrhage; transfusion requirement; DIC; hypovolemic shock
Surgical	Urologic injury (bladder, ureter); gastrointestinal injury; vascular injury; nerve injury
Postoperative	Infection (wound, pelvic abscess, sepsis); venous thromboembolism (DVT/PE); wound dehiscence
Reproductive/long-term	Loss of fertility (hysterectomy); intra-abdominal adhesions; chronic pelvic pain
Maternal outcome	Increased morbidity: prolonged ICU stay; increased mortality risk
Neonatal outcome	Preterm birth; low birth weight; NICU admission; neonatal mortality

DIC: disseminated intravascular coagulation; DVT: deep venous thrombosis; ICU: intensive care unit; NICU: neonatal intensive care unit; PAS: placenta accreta spectrum; PE: pulmonary embolism.

Common complications of hysterectomy generally reported include infection (9-13%), thromboembolism (1-12%), genitourinary injury (1-2%), gastrointestinal injury (0.1-1%), and bleeding (median blood loss 156 - 660 mL) [7]. Yared et al [8] reported bladder or ureteral injuries in 32% of hysterectomy cases, recommending multidisciplinary care and prophylactic ureteral stenting to reduce morbidity. Pinto et al [9] found hysterectomy was required in 11.2% of PAS cases, while urologic injuries occurred in 8.8%.

Hessami et al published an interesting meta-analysis. This meta-analysis of 2,300 women with PAS found that cesarean hysterectomy led to higher blood loss, more transfusions, and increased genitourinary injuries compared to conservative approaches, though ICU admission and thromboembolic risks were like some conservative methods. Conservative management, including placenta left *in situ* or local resection, was associated with lower surgical morbidity and may preserve fertility. These findings support conservative strategies as a viable alternative to cesarean hysterectomy, but further randomized and long-term studies are needed to confirm safety and outcomes [10].

Vatanchi et al [11] observed urinary tract injuries in 25% of PAS hysterectomies, mainly cystotomy, with many developing overactive bladder symptoms. Orsi et al [12] reported a 17.7% rate of urological complications, mainly bladder injuries, associated with prior cesareans and high blood loss.

Vascular injury risk increases with obesity, adhesions, anatomical variations, and low surgical volume [13, 14]. Levin et al [15] found vascular repair was needed in 0.09% of 201,224 gynecologic surgeries, mostly during hysterectomy. Lopez-Vera et al [16] noted 4.5 hysterectomies per 1,000 obstetric procedures, with selective arterial ligation used in 88% of cases.

Bleeding remains a major concern in PAS. Jurkovic et al [17] reported significant bleeding in 38% of cesarean scar pregnancies (CSPs) managed beyond 12 weeks. Definitions of “severe bleeding” vary across studies, complicating data comparison [18-20].

During surgery, the patient experienced uncontrolled hemorrhage, vascular injury, and bladder damage - events that rapidly escalated the clinical complexity. The timely conversion from spinal to general anesthesia (intraoperatively) was a pivotal decision, enabling airway control, improved hemodynamic management, and facilitation of prolonged surgical intervention. Early recognition of the surgical challenges prompted immediate involvement of a vascular surgeon and a urologist, ensuring rapid hemostasis and bladder repair. Activation of the MTP was triggered by sustained bleeding and hemodynamic instability, allowing timely replacement of blood products and correction of coagulopathy. This coordinated, multidisciplinary response, guided by clear communication between anesthesiology, surgery, and transfusion teams, was instrumental in restoring stability and achieving a favorable outcome despite limited resources.

Massive obstetric hemorrhage most often from placental disorders or uterine atony, remains a leading cause of maternal mortality. Delays in resuscitation and surgical control are key contributors to poor outcomes. Early volume resuscitation, blood transfusions, and uterotonics were vital in this case [21]. Definitive surgical control was achieved through vessel ligation and hysterectomy.

Massive hemorrhage (typically > 2,500 mL) requires MTP activation and early fibrinogen replacement [22]. The RCOG defines “massive” antepartum hemorrhage as blood loss > 1,000 mL or any bleeding causing shock [23]. Greene et al [24] observed a 54% increase in major bleeding between 2011 and 2018, with median losses > 3,000 mL.

ACOG recommends cesarean delivery for PAS with active bleeding regardless of gestation, favoring expectant management only if bleeding ceases before 36 weeks [25]. In a review of 62 MTP cases, PAS (32%) and atony (34%) were leading causes, with favorable survival outcomes [26]. Balanced transfusion ratios (∼1:1:1 for PRBCs/FFP/platelets) improved hemostasis and reduced coagulopathy [27, 28]. Anesthetic principles of massive bleeding management are summarized in Table 2.

**Table 2:** Anesthetic Management of Massive Bleeding [21-28]

Step	Intervention	Description
1	Monitoring and access	Establish large-bore IV access (two lines), arterial line, and central venous access if needed. Continuous hemodynamic and urine output monitoring.
2	Airway and oxygenation	Early airway control with intubation if patient unstable or anticipated rapid deterioration. Provide 100% oxygen.
3	Volume resuscitation	Start with balanced crystalloids, transition quickly to blood products. Apply massive transfusion protocol (1:1:1 ratio of RBC/FFP/platelets).
4	Hemostatic agents	Administer tranexamic acid early (within 3 h), consider fibrinogen concentrate or cryoprecipitate if hypofibrinogenemia present.
5	Anesthetic technique	GA in unstable patients; regional may be used in selected stable cases (but be prepared to convert to GA).
6	Adjunctive measures	Maintain normothermia, correct acidosis, optimize calcium levels during transfusion, permissive hypotension
7	Team communication	Close coordination with obstetricians, blood bank, and ICU team. Activate massive transfusion protocol early.
8	Postoperative care	Transfer to ICU for ongoing resuscitation, monitoring, and correction of coagulopathy.

GA: general anesthesia; ICU: intensive care unit; IV: intravenous; RBC: red blood cell; FFP: fresh frozen plasma.

Studies support a 1:1 FFP/RBC ratio for optimal hemostasis [29]. Panigrahi et al [30] found higher transfusion needs in increta/percreta (82%) than accreta (71%). Balanced resuscitation strategies, including TXA within 3 h, reduce coagulopathy and mortality [31-36]. The WOMAN trial was a randomized, double-blind study conducted across 193 hospitals in 21 countries, enrolling 20,060 women with postpartum hemorrhage who were assigned to receive either TXA or placebo. TXA significantly reduced death due to bleeding (1.5% vs. 1.9%), particularly when administered within 3 h of birth, but it did not reduce hysterectomy rates or the composite outcome of death or hysterectomy. Adverse events, including thromboembolic complications, were comparable between groups [37].

Maintaining an MAP around 50 mm Hg without hypoperfusion may also limit blood loss [38, 39]. The actual strategies to reduce massive bleeding in obstetrics are summarized in Table 3.

**Table 3:** Our Practice to Reduce Massive Bleeding in Obstetrics

Intervention	Description
Uterotonics and uterine massage	First-line measures to stimulate uterine contraction and reduce bleeding.
Tranexamic acid	Early administration (within 3 h) to reduce fibrinolysis and improve survival.
Volume replacement	Use crystalloids and initiate massive transfusion protocols (1:1:1 ratio).
Surgical interventions	Balloon tamponade, compression sutures, arterial ligation, or hysterectomy if conservative methods fail.
Correction of coagulopathy	Administer FFP, platelets, cryoprecipitate, or fibrinogen concentrate as indicated.
Hemodynamic	Permissive hypotension
Multidisciplinary approach	Close coordination between obstetric, anesthetic, surgical, and blood bank teams.

FFP: fresh frozen plasma.

The procedure began under spinal anesthesia, as initially planned by the obstetric team, with two large-bore IV accesses. When uncontrolled bleeding developed, rapid conversion to general anesthesia was essential to secure the airway and optimize hemodynamic control. Central venous and arterial lines were promptly inserted, and aggressive volume resuscitation was initiated, followed by activation of the MTP. The patient received 14 units of PRBCs, 10 units of FFP, 12 platelet units, 2 g of TXA, and continuous norepinephrine infusion (0.05 - 0.2 µg/kg/min).

Continuous monitoring of hemoglobin, lactate, and perfusion indices along with meticulous prevention of hypocalcemia and hypothermia guided intraoperative management. Despite the absence of advanced coagulation tools, clinical assessment and structured team coordination enabled effective hemostasis and hemodynamic stabilization. The patient was transferred to the ICU extubated and remained stable throughout recovery.

This case was managed in a resource-limited setting, where comprehensive coagulation monitoring was not feasible due to the unavailability of ROTEM and fibrinogen concentrate, requiring empirical initiation of the MTP. While the outcome was favorable, the single-case nature of this report and the limited postoperative follow-up restrict the generalizability of our observations. Despite these limitations, the case highlights the importance of early recognition, prompt activation of MTPs, rapid anesthetic adaptation, and coordinated multidisciplinary care in achieving successful outcomes in high-risk PAS patients, even when advanced diagnostic or monitoring tools are not available.

### Conclusions

Effective management of massive obstetric hemorrhage requires prompt identification of the bleeding source and timely interventions tailored to the patient’s condition. In this case, early activation of the MTP allowed rapid replacement of blood loss with balanced blood products, while TXA was administered to support hemostasis. The decision to convert from spinal to general anesthesia was guided by hemodynamic instability and surgical complexity, enabling safe completion of hysterectomy and bladder repair. Targeted surgical measures, including compression sutures and careful hemostatic techniques, were applied when conservative methods were insufficient. Close multidisciplinary communication between obstetric, anesthetic, surgical, and critical care teams, combined with immediate blood bank support, was critical in preventing maternal morbidity and achieving a favorable outcome.

### Learning points

1) Early activation of the MTP with a balanced 1:1:1 ratio and prompt use of TXA are vital to control hemorrhage and prevent coagulopathy in PAS.

2) Timely anesthetic conversion to general anesthesia and multidisciplinary coordination among obstetric, anesthetic, surgical, and transfusion teams are essential for optimal intraoperative management.

3) Vigilant monitoring and correction of hypothermia, hypocalcemia, and acidosis, can achieve favorable outcomes even in resource-limited settings.

## Data Availability

The data supporting the findings of this study are available from the corresponding author upon reasonable request.
